# Cases of *Echinococcus granulosus* Sensu Stricto Isolated from Polish Patients: Imported or Indigenous?

**DOI:** 10.1155/2015/728321

**Published:** 2015-09-30

**Authors:** Monika Dybicz, Piotr Karol Borkowski, Julia Dąbrowska, Lidia Chomicz

**Affiliations:** ^1^Department of General Biology and Parasitology, Medical University of Warsaw, 5 Chałubińskiego Street, 02-004 Warsaw, Poland; ^2^Department of Zoonoses and Tropical Diseases, Medical University of Warsaw, 37 Wolska Street, 01-201 Warsaw, Poland; ^3^Department of Medical Biology, Medical University of Warsaw, 73 Nowogrodzka Street, 02-018 Warsaw, Poland

## Abstract

The cases of nine Polish patients with diagnosed cystic echinococcosis (CE) were examined. A total of nine isolates obtained postoperatively were investigated using PCR and sequencing. The mitochondrial region of *nad1* gene was amplified. This PCR and sequencing analysis revealed the presence of *Echinococcus canadensis* G7 in seven patients and *E. granulosus* G1 in two patients. These data demonstrate that *E. canadensis* is the predominant causative agent of human cystic echinococcosis in Poland. *E. granulosus* G1 detection in Polish patients suggests that the parasite was imported; however it does not exclude the possibility that these cases could have been of Polish origin.

## 1. Introduction

Cystic echinococcosis (CE) is caused by the metacestode stage of the* Echinococcus granulosus* complex belonging to the family Taeniidae. CE is considered as one of the most important zoonotic parasitic diseases worldwide. The life cycle of* Echinococcus* species is mostly domestic, involving dogs as the definitive host, in which the adult worm lives in the small intestine. Wild canids (dingoes, wolves, jackals, coyotes, red foxes, etc.) can also serve as definitive hosts in the transmission cycle. The hydatid cyst metacestode develops in the internal organs of an intermediate host that acquires the infection through accidental ingestion of the tapeworm eggs. The intermediate hosts can be sheep, goats, swine, cattle, and humans. The cysts in humans develop mainly in the liver (70%), lungs (20%), and other organs like the brain, heart, and bones [1]. The cysts usually may develop asymptomatically for years and clinical symptoms occur when the cysts press on the surrounding tissues or organs. CE can be life-threatening when the cysts rupture into the peritoneal cavity causing anaphylaxis or can cause secondary CE. Echinococcosis is regarded as an emerging disease occurring worldwide with the highest prevalence in parts of Eurasia, north and east Africa, Australia, and South America [2–6].

Analysis of mitochondrial and nuclear genes of different* Echinococcus* species has led to taxonomic revisions and the genotypes G1–G3 are now grouped as* E. granulosus* sensu stricto, G4 is as* Echinococcus equinus*, G5 is as* Echinococcus ortleppi*, G6–G10 are as* Echinococcus canadensis*, and the “lion strain” is as* Echinococcus felidis* [7–11]. Among these strains,* E. granulosus* sensu stricto has a broad geographical distribution with a wide host range and is the major causative agent of human cystic echinococcosis.

In Poland, identified CE human cases have not been common; 260 patients have been treated in the Department of Zoonoses and Tropical Diseases within 10 years. The main* Echinococcus* strain, detected in Polish domesticated animals (pigs, dogs), is* E. canadensis* (G7) [12]. Molecular studies of the cysts isolated from humans revealed the presence of the G7 genotype [12, 13].* E. canadensis* G7 strain has also been identified in humans in Ukraine [12], the Slovak Republic [12, 14], Turkey [15], and Austria [16]. Recently the first human cases of G7 infection in Mongolia [17], South Africa [18], and China [19] were found.

In our report we aim to characterize species of* Echinococcus* causing CE among cases of patients who underwent surgery to remove a cyst.

## 2. Materials and Methods

### 2.1. Materials

Fragments of cysts were collected from 9 patients postoperatively, carried out between January 2010 and May 2015 in the Department of Zoonoses and Tropical Diseases of the Medical University of Warsaw, Poland. Some patients treated with albendazole due to having cystic echinococcosis have been under medical supervision for many years. On account of some complications the cysts were removed surgically. The patient data collected included age, gender, and city. Six patients were females and three were males. The age of the patients ranged from 26 to 77 years. The examined samples represented by part of a cyst were stored frozen at −20°C or fixed in 70% ethanol prior to molecular analysis.

### 2.2. DNA Extraction and PCR

The samples of examined isolates and* E. canadensis* G7 positive control (isolates JX266793 and JX266824) were rinsed with phosphate-buffered saline (PBS) several times to remove any ethanol and centrifuged at 5000 ×g for 10 min. Each pellet was dissolved in 100 *μ*L PBS and genomic DNA was extracted using a NucleoSpin kit (Macherey-Nagel, Düren, Germany) following the manufacturer's instructions. Two mitochondrial regions were amplified by PCR using the prepared DNAs as templates. Part of the NADH dehydrogenase 1 (*nad1*) gene was amplified using primers JB11 (5′-AGATTCGTAAGGGGCCTAATA-3′) and JB12 (5′-ACCACTAACTAATTCACTTTC-3′) [20]. The 50 *μ*L reactions were comprised of 1 *μ*L of DNA template, 50 pM of each primer, 0.2 mM of each dNTP, 1x PCR buffer containing 2.5 mM MgCl_2_, and 1 U of Taq DNA polymerase (Qiagen, Hilden, Germany). The following PCR was performed in a PTC-200 thermal cycler (MJ Research, Waltham, USA) in conditions: 3 min at 95°C followed by 35 cycles of 1 min at 95°C, 1 min at 50°C, and 1 min at 72°C. The PCR products were separated by electrophoresis on a 2% agarose gel (MetaPhor, FMC BioProducts, Philadelphia, USA) and then stained with ethidium bromide and observed on a UV transilluminator. The* nad1* gene fragments were purified and then directly sequenced in both directions using a BigDye Ready Reaction Cycle Sequencing kit and an ABI 3730 Genetic Analyzer (Applied Biosystems, Foster City, USA). Chromatograms were manually checked and edited using Chromas 2.0. The obtained sequences were aligned with others retrieved from NCBI GenBank using ClustalW2 (http://www.ebi.ac.uk/Tools/clustalw2).

## 3. Results

A total of 9 samples were obtained from patients who underwent surgery to remove a cyst located in the liver (6 cases of women and 2 men) and in the hip muscle (1 case of a man). The patients came mostly from Warsaw, other cities and villages of central Poland, where they lived in either urban (40%) or rural areas (60%), and most of them had close contact with dogs. Eight patients suffered from primary and one patient from secondary cystic echinococcosis. The majority of the cysts ranged in size from 3 to 7 cm in diameter, and the largest one was 9 cm; 6 out of 9 cases were active (mostly type CE2), whereas the remaining cysts were sterile (type CE3) (Table 1). DNA extracted from all 9 samples and positive controls were used as the template in separate PCRs to amplify region of the mitochondrial NADH dehydrogenase 1. Each PCR produced a single band upon agarose gel electrophoresis. All isolates and controls were diagnosed as positive by amplification of* nad1* fragment (~500 bp). The* nad1* fragments were sequenced and compared with sequences of* Echinococcus* genotypes available in NCBI GenBank. The sequences of 7 isolates showed 100% identity to that of the pig strain G7, designated* E. canadensis* (Table 1). The sequences of 2 other isolates were identical to the sheep strain* E. granulosus* G1 (Table 1). All* nad1* sequences were deposited in GenBank with accession numbers KT780293–KT780301.

## 4. Discussion

*E. granulosus* sensu lato is a complex of genetic variants with genotypic and phenotypic differences such as intermediate host specificity or development. Recent molecular studies on the taxonomy of* E. granulosus* sensu lato have revealed that it is a complex of five independent species [7–11]. The genetic identification has been significant in understanding the transmission of the parasite between definitive and intermediate hosts, including humans. The cosmopolitan strain of* E. granulosus* is the G1 (sheep) genotype distributed worldwide and occurring particularly in areas of extensive sheep farming. It is the predominant strain infecting humans, but the other genotypes are also known to be infective. The presence of other* Echinococcus* strains in humans has been confirmed in Argentina, Kenya, Egypt (G1, G2, G5, and G6), Poland, Ukraine, the Slovak Republic, Turkey, Austria, South Africa (G7), Russia, and Mongolia (G10) [12–18, 21–25]. Transmission of* E. canadensis* G7 is considered to be restricted to certain areas in central, southern, and eastern Europe, Spain, Poland, Lithuania, the Slovak Republic, Ukraine, Romania, and Italy [12–16, 26–30], where the most common intermediate host is the pig. Recently,* E. canadensis* G7 has also been identified in pigs from Mexico and Brazil [31–33]. It has often been assumed that* Echinococcus* strains isolated from pigs of European origin are a separate strain and have low infectivity for humans and domestic ungulates [34]. Genetic investigations based on* nad1* sequence alignment showed the presence of* E. canadensis* G7 in Polish patients [12, 13], although another pig strain identified in human was designated G9, closely related to G6 (based on* its1* region analysis) and to G7 (*nad1* sequence analysis) [35].

In this report, we used PCR and sequencing to characterize* Echinococcus* cysts isolated from the liver and hip muscle of patients who underwent surgery to remove the cyst during the period of the last 5 years. In this analysis samples from 9 patients were examined. The patients were diagnosed as having cystic echinococcosis, following histopathological examination, imaging techniques, and/or immunological tests. Most of these patients have been under medical supervision for many years and treated with albendazole for months and due to some complications (bleeding inwards, detachment of internal capsule, and strong abdominal pain) it was decided that the cyst should be removed. Of these 9 samples, we were able to diagnose 7 cases as liver CE caused by* E. canadensis*, predictably as it was described already in a group of 30 patients in Poland [13].

The* nad1* sequences from 7 samples corresponded to the* E. canadensis* G7 strain. Taking into consideration the fact that* E. canadensis* G7 has already been confirmed in Poland and pigs are the major intermediate host, the G7 strain plays a significant role being the aetiological agent of human cystic echinococcosis in Poland. Interestingly, 2 other samples were identical to* E. granulosus* G1. This is the first report revealing* E. granulosus* sensu stricto hydatids isolated from humans carried out in Poland. These isolates involved a female and a male. The female patient (case number 6) with diagnosed CE of the liver, resembling sheep strain in computer tomography, was treated with albendazole. The cyst had two daughter cysts and after albendazole treatment, detachment of the capsule and parasite death were observed. A year later the patient suffered from very strong abdominal pain and the deceased hydatid was surgically excised. In the interview with the patient it was denoted that she travelled to Turkey a few years ago and spent a week there before CE was diagnosed, suggesting possibility of infection with G1 strain abroad. The Polish male patient (case number 8) was operated on in 2001 in Kazakhstan on account of the cyst found in the muscle of the left hip but the diagnosis of CE was not confirmed by histopathological or any other study. At present, after resettlement to Poland, colon cancer has been diagnosed and during routine tomography small cysts have been localized in the left hip muscle. The tumor and the cysts were removed and sent for confirmation. The preliminary identification of* E. granulosus* G1 in this case, considering the patient's history, implies the case has been imported from Kazakhstan where G1 strain is predominant. This fact does not exclude the possibility of infection of different hosts by* E. granulosus* G1 or other strains in Poland. The knowledge of the distribution of* Echinococcus* genetic variants in all parts of the country has not been ascertained yet.

Genotypes* E. granulosus* G1 and* E. canadensis* G7 are morphologically and genetically different; however both can develop in humans and pigs, generating threats to the public health and the animal breeding. Therefore, it is necessary to examine the pathology and etiology of CE based on molecular identification to assess the genotype. Still many aspects remain inadequately determined such as the factors establishing host specificity and developmental diversity among different strains of* Echinococcus*.

## 5. Conclusion

Cystic echinococcosis is an important zoonotic emerging disease occurring worldwide which is caused by the hydatid cyst metacestode stage of different* Echinococcus* species. In Poland, human CE is considered to be rather infrequent and mainly induced by the G7 pig strain of* E. canadensis*. In this report we identified* E. canadensis* G7 in seven human liver cysts; however in two cases (liver and hip muscle) the hydatids were diagnosed as* E. granulosus* G1. As it follows,* E. canadensis* has been the predominant strain in Poland, but revealing G1 strain in two patients remains unclear if these have been imported cases or maybe indigenous ones.

## Figures and Tables

**Table 1:** The characterization of nine CE cases, including patient data, diagnosis, and species. F: female, M: male.

Case number	City/region	Age in years, sex	Diagnosis details	Species
1	Warsaw (central Poland)	55 F	Liver single cyst (~6 cm), type CE2, removed with albendazole administration.	*E. canadensis* G7

2	Central Poland	77 M	Liver single cyst (3.5 cm), type CE2, removed with albendazole administration.	*E. canadensis* G7

3	Central Poland	48 F	Three cysts in the liver (size 5–7 cm), type CE2, removed with albendazole administration.	*E. canadensis* G7

4	Warsaw (central Poland)	31 F	Single liver cyst (~5 cm), type CE3, treated with albendazole for months and then removed. ELISA and Western blot positive.	*E. canadensis* G7

5	Ostrołęka (northeast of central Poland)	26 F	Single liver cyst (~9 cm), type CE2. When bleeding occurred, the cyst was removed with albendazole administration.	*E. canadensis* G7

6	Warsaw (central Poland)	28 F	Single cyst (6.5 cm), type CE2 with two cysts inside (1.4 and 2.2 cm), located at the surface of liver, not treated pharmacologically, Western blot positive. When severe abdominal pain occurred, cyst was removed. Patient spent one week in Turkey 2 years before CE was diagnosed.	*E. granulosus* G1

7	Central Poland	53 F	Single liver cyst (~5 cm), type CE3, removed with albendazole administration.	*E. canadensis* G7

8	Warsaw (central Poland)	60 M	Three cysts (each ~4 cm) in the hip muscle, type CE2. The cysts were detected during CT scan of colon cancer diagnosis and then removed. In 2001, the primary cyst from hip muscle was removed in Kazakhstan.	*E. granulosus* G1

9	Central Poland	48 M	Single liver cyst, removed with albendazole administration.	*E. canadensis* G7
